# Changes in Body Temperature Patterns Are Predictive of Mortality in Septic Shock: An Observational Study

**DOI:** 10.3390/biology12050638

**Published:** 2023-04-22

**Authors:** Benjamin Coiffard, Hamid Merdji, Mohamed Boucekine, Julie Helms, Raphaël Clere-Jehl, Jean-Louis Mege, Ferhat Meziani

**Affiliations:** 1 IHU-Méditerranée Infection, IRD, AP-HM, MEPHI, Aix Marseille Université, 13005 Marseille, France; jean-louis.mege@univ-amu.fr; 2 Médecine Intensive-Réanimation, APHM, Hôpital Nord, Aix Marseille Université, 13015 Marseille, France; 3 Service de Réanimation, Nouvel Hôpital Civil, Université de Strasbourg (UNISTRA), Faculté de Médecine, Hôpitaux Universitaires de Strasbourg, 67000 Strasbourg, France; hamid.merdji@chru-strasbourg.fr (H.M.); julie.helms@chru-strasbourg.fr (J.H.); raphael.clere-jehl@chru-strasbourg.fr (R.C.-J.); ferhat.meziani@chru-strasbourg.fr (F.M.); 4 UMR 1260, Regenerative Nano Medecine, INSERM, Fédération de Médecine Translationnelle de Strasbourg (FMTS), Université de Strasbourg, 67000 Strasbourg, France; 5 Health Service Research and Quality of Life Center, APHM, EA 3279 CEReSS, School of Medicine-La Timone Medical Campus, Aix Marseille Université, 13005 Marseille, France; boucekine.m@gmail.com

**Keywords:** circadian rhythm, temperature, septic shock, intensive care units, chronobiology disorders, mortality

## Abstract

**Simple Summary:**

We applied, here, mathematical modeling to the body temperature time series (sinusoidal regression and cosinor analysis) in a cohort of septic shock to describe an accurate temperature course and its relation to mortality. Lower mesor (mean temperature) and higher amplitude were associated with mortality and could be considered an interesting prognostic marker in septic shock. In the age of artificial intelligence, the incorporation of such data in an automated scoring alert could compete with physicians to identify high-risk patients during septic shock. Besides, we demonstrated that many factors, such as specific therapeutic strategies, may influence the normal body temperature rhythm.

**Abstract:**

Biological rhythms are important regulators of immune functions. In intensive care unit (ICU), sepsis is known to be associated with rhythm disruption. Our objectives were to determine factors associated with rhythm disruption of the body temperature and to assess the relationship between temperature and mortality in septic shock patients; In a cohort of septic shock, we recorded body temperature over a 24-h period on day 2 after ICU admission. For each patient, the temperature rhythmicity was assessed by defining period and amplitude, and the adjusted average (mesor) of the temperature by sinusoidal regression and cosinor analysis. Analyses were performed to assess factors associated with the three temperature parameters (period, amplitude, and mesor) and mortality. 162 septic shocks were enrolled. The multivariate analysis demonstrates that the period of temperature was associated with gender (women, coefficient −2.2 h, *p* = 0.031) and acetaminophen use (coefficient −4.3 h, *p* = 0.002). The mesor was associated with SOFA score (coefficient −0.05 °C per SOFA point, *p* = 0.046), procalcitonin (coefficient 0.001 °C per ng/mL, *p* = 0.005), and hydrocortisone use (coefficient −0.5 °C, *p* = 0.002). The amplitude was associated with the dialysis (coefficient −0.5 °C, *p* = 0.002). Mortality at day 28 was associated with lower mesor (adjusted hazard ratio 0.50, 95% CI 0.28 to 0.90; *p* = 0.02), and higher amplitude (adjusted hazard ratio 5.48, 95% CI 1.66 to 18.12; *p* = 0.005) of temperature. Many factors, such as therapeutics, influence the body temperature during septic shock. Lower mesor and higher amplitude were associated with mortality and could be considered prognostic markers in ICU. In the age of artificial intelligence, the incorporation of such data in an automated scoring alert could compete with physicians to identify high-risk patients during septic shock.

## 1. Introduction

The interest in circadian rhythms is growing because of their crucial role in homeostasis, as evidenced by the 2017 Nobel Prize, attributed to the discovery of clock genes, which generate biological rhythms [1,2,3]. Moreover, circadian rhythms are now recognized as important regulators of host defense functions, and the relationship between circadian disruption and immune dysfunction is now recognized [4,5]. In the intensive care unit (ICU), several factors, such as stress, light/dark disturbance, sedation, and systemic inflammation, might be responsible for circadian rhythm alterations [6]. Several studies have studied core body temperature (CBT) rhythmicity as a marker of the biological clock during the ICU stay [7,8]. ICU patients are likely to have a disrupted circadian rhythm of body temperature, thereby maybe compromising biological function efficiency and recovery [9,10]. The CBT, which varies diurnally by approximately 0.5 °C around a mean of 37.0 °C in healthy individuals, is considered a physiological marker of the circadian clock [3]. CBT measurement is one of the oldest clinical tools available, and fever remains a common indicator of illness, especially infection [11]. 

In mammals and humans, circadian rhythms are controlled by a central circadian pacemaker located in the suprachiasmatic nuclei (SCN) of the hypothalamus [2,3]. The circadian clock cannot be measured directly in humans, and surrogate markers have been used to measure its output, such as cortisol and melatonin [12,13]. The circadian component of CBT is under the control of SCN, which then transmits signals to the thermoregulation centers. While the thermoregulatory system acts to maintain the temperature at a fixed target value, this set point fluctuates during the day, resulting in circadian variations [14]. Body temperature cycles have recently been demonstrated to function as systemic cues that efficiently participate in the synchronization of peripheral clocks in mammals [15,16]. 

Sepsis is a common problem in critically ill patients and immunosuppression caused by physiologic stress such as circadian rhythm disruption might play a negative role in the host’s response. In sepsis, fever generation occurs through several mechanisms, including cytokine production, and it may be associated with mortality [17]. To date, most physicians focus on the presence or absence of fever, rather than following temperature trends. However, increasing evidence suggests that variability in the patterns of physiologic measurements, such as heart rate or body temperature, may be more specific to infection and might be an earlier indicator of sepsis than standard diagnostic criteria [18,19]. 

The objectives of this observational study were to determine factors associated with rhythm disruption of the CBT and to assess the relationship between temperature and mortality in septic shock patients. 

## 2. Materials and Methods

### 2.1. Study Design, Patient Selection 

All consecutive adult patients (18–85 years old) admitted to our medical intensive care unit (Strasbourg University Hospital) between July 2013 and February 2016 for septic shock [20] and treated with norepinephrine and/or epinephrine after fluid challenge were enrolled. Patients with end-stage chronic diseases or DNR (do not resuscitate) options were excluded. The visitation policy of our facility is an open policy that always allows family access (24 h), with a restriction on the number of family members. Our ICU’s policy requires nurses to turn off the light after dark, except when emergency procedures are required. As suggested by the World Health Organization, our ICU’s policy strongly suggests to the medical and paramedical team, but also to visitors, to limit noise levels below 35 dB–40 dB [21]. The ambient ICU temperature is automatically set between 20 °C and 22 °C through a thermostat. In accordance with the guidelines, enteral nutrition was initiated at a low dose within 12–24 h of admission to the ICU for most patients [22]. In the initial 24-h period, a caloric target of 700 kcal per day was pursued, which equates to an enteral feeding solution infusion rate of 21 milliliters per hour of a 1.4 kcal per milliliter solution.

### 2.2. Temperature Measurement

The temperature was measured 24 h on day 2 after admission, according to local practices whether with central devices in the bladder, esophagus, or intravascular probes (Mon-a-Therm Foley Catheter with Temperature Sensor 400TM, Covidien, Dublin, Ireland) every 2 h or by tympanic measurement (Genius 3 Tympanic Thermometer, Covidien, Dublin, Ireland) every 4 h when no central monitoring was available. These devices are compliant with standards ISO and IEC and have already been validated in numerous studies assessing their accuracy [23,24].

### 2.3. Body Temperature Rhythm Analysis

The first part of the analysis consisted of assessing the individual time-period of the temperature (the “period” is the time needed for one complete cycle of temperature). This analysis was performed with the PAST3 software (URL https://palaeo-electronica.org/2001_1/past/issue1_01.htm accessed on 21 June 2001) [25]. According to the author’s description, the “fitted periods” were obtained by the matching pursuit algorithm by sequentially optimizing the period of each sinusoid (over the full meaningful range from one period to the Nyquist frequency), after subtracting all previously fitted sinusoids. 

In the second part, a single cosinor analysis with R software (package cosinor) was performed for each patient to obtain the other rhythm parameters (i.e., mesor and amplitude) [26]. This analysis uses the least-squares method to fit a sine wave to a time series with the model specified as:

y = mes + amp × cos(2π(t − ϕ)/period)

where y was the marker value (temperature), t represented time-of-day in decimal hours, mes represented the mesor, amp the amplitude, ϕ the acrophase of the rhythm, and period the fit period obtained in the first part. The MESOR (Midline Estimating Statistic Of Rhythm) represents the mean of the modeled rhythm over the time studied. The amplitude is the difference between mesor and the peak value.

### 2.4. Statistical Analysis

Data were analyzed using the public software R version 4.2.1; R Development Core Team (2005) (R: A language and environment for statistical computing. R Foundation for Statistical Computing, Vienna, Austria. ISBN 3-900051-07-0, URL: http://www.R-project.org, accessed on 20 June 2022). Figures were performed using the R package ggplot2. For all tests, the statistical significance was defined as a *p*-value < 0.05. Continuous variables were expressed as mean values (±standard deviation) or median values [interquartile], and comparisons between two groups were performed using Student’s *t*-test or the Mann-Whitney test, according to the distribution (Shapiro-Wilk test). Discrete variables are expressed as percentage values, and comparisons between groups were performed using a chi-squared test. The relations between the temperature parameters (i.e., period, mesor, and amplitude) and ICU variables were analyzed by the general linear model, and the regression coefficients ß ± standard error (SE) were reported to compare the relative predictive effects of the independent variables. Survival analysis was performed using Cox proportional risk model to determine the factors associated with 28-day mortality. Relative risks, the hazard ratio (HR), and their 95% confidence intervals (95% CI) were calculated. All variables with *p* < 0.05 values in univariate models were included in the final multivariate models. The 28-day survival between groups was calculated by the log-rank test, and survival curves were obtained by the Kaplan-Meier method. To form groups with the highest survival difference, the best significant threshold of the temperature parameter for predicting 28-day mortality was calculated with the cutp function in R to determine the optimal cut point for a continuous variable in the Cox model.

## 3. Results

### 3.1. Population Characteristics

During the study period, 162 septic shock patients were included with a median age of 65 years, interquartile range 56–74 years, and a mortality rate of 23% in the ICU (Table 1). The lung (46%) was the most frequent site of infection. Infection was microbiologically documented in 128 (79%) patients, and the percentage of bacteremia was 44%. Enterobacteria, Streptococcus, and Staphylococcus were the most frequent agents (Table S1). The most common comorbidity was diabetes in 51 of the cases (31%). An amount of 141 (87%) patients were mechanically ventilated for a median duration of six days [3,4,5,6,7,8,9,10,11,12,13]. On day 2, a majority received sedation (68%), vasopressor support (77%), and substitution with hydrocortisone (57%).

### 3.2. Heterogeneity of the Temperature Rhythm

**Figure 1:**
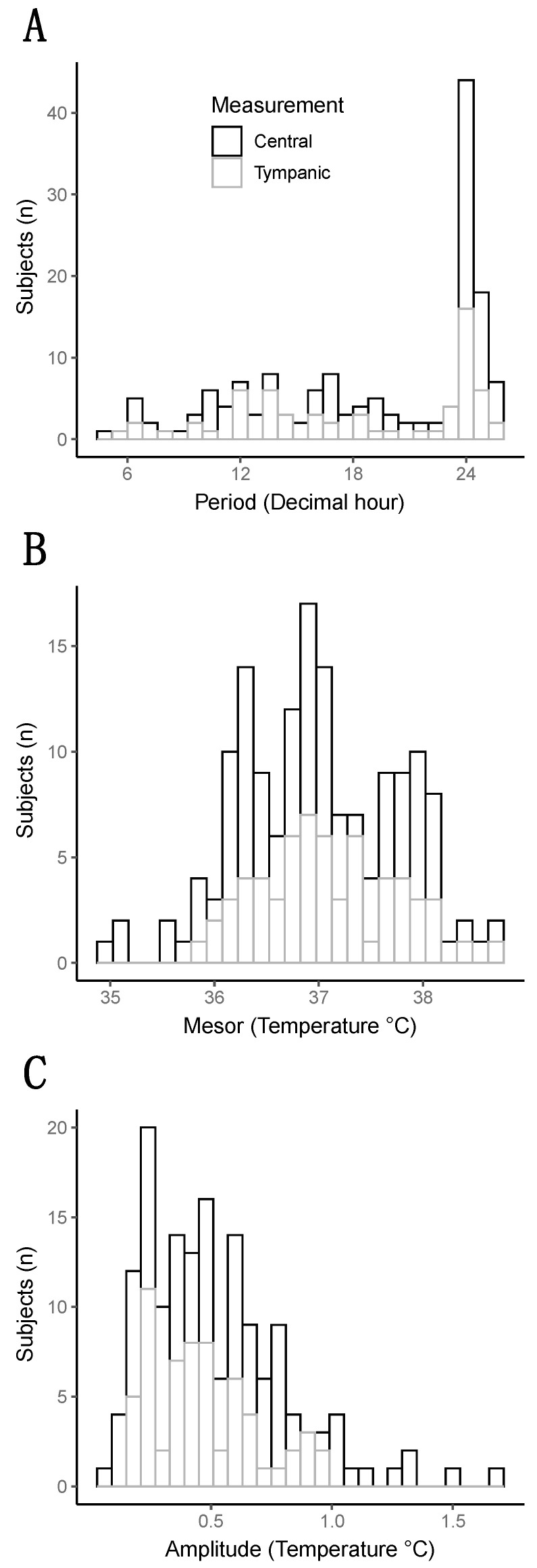
Distribution of the three parameters of the temperature (Period, Mesor, and Amplitude). (**A**) Histogram representing the distribution of the period (decimal hour). (**B**) Histogram representing the distribution of the mesor (degree Celsius). (**C**) Histogram representing the distribution of the amplitude (in degree Celsius).

The rhythm of CBT in the whole cohort was close to the physiological circadian rhythm of the temperature of 37 degrees with an amplitude of 0.5 degrees Celsius. However, the large distribution of these parameters revealed a huge heterogeneity between patients (Figure 1).

**Figure 2:**
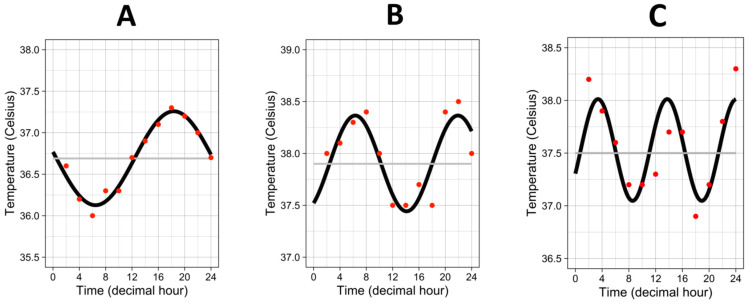
Three examples of temperature rhythm. Red dots represent the raw values of temperature from one individual. Black curves represent the cosinor curves fitted to the data of the individual. Grey lines represent the mesors. (**A**) period = 23.8 h, amplitude = 0.57 °C, mesor = 36.7 °C. (**B**) period = 15.6 h, amplitude = 0.46 °C, mesor = 37.9 °C. (**C**) period = 10.35 h, amplitude = 0.48 °C, mesor = 37.5 °C.

The median “period” (i.e., the time needed for one complete cycle of temperature) was 20.8 h, interquartile range 13.7–24.1 h. Half of the patients showed a “period” close to 24 h, but the other half exhibited a faster rhythm with a “period”, which could go down to 4 h to a complete cycle of temperature, as attested by the three examples in Figure 2. 

The “mesor”, which represents an estimation of the average temperature by the mathematical analysis, was, at mean, 36.99 degrees +/−0.75. The “amplitude”, which is the difference between the peak value of the temperature and the “mesor”, had a median of 0.46 degrees [0.28–0.67] in the whole cohort.

### 3.3. Factors Associated with the Body Temperature Parameters

As the profiles of temperature rhythm were extremely different between patients, we assessed factors influencing the three parameters (i.e., period, mesor, and amplitude) of the temperature: the patient’s characteristics, infections, and therapies used at the time of temperature measurement (day 2) to identify factors associated with circadian disruption (Table S2). 

Multivariate regression analysis revealed significant associations: a lower period in women (coefficient at −2.2 h, SE 0.99), as well as in patients treated with acetaminophen (coefficient at −4.3 h, SE 1.4). Analysis of mesor demonstrated a negative correlation between SOFA score and the temperature (coefficient at −0.05 degree per SOFA point, SE 0.02), as well as between hydrocortisone and temperature (coefficient at −0.5 degree, SE 0.16). A positive correlation between procalcitonin and temperature mesor was found (coefficient at 0.004 degrees per ng/mL, SE 0.001). Analysis of amplitude demonstrated lower amplitude in patients treated with renal replacement therapy, coefficient at −0.49 degree, SE 0.16 (Table 2).

### 3.4. Low Mesor and High Amplitude Were Associated with 28-Day Mortality

In this part, we performed a survival analysis to assess if the rhythm of the temperature was associated with sepsis mortality on day 28. A univariate Cox regression analysis was performed to assess factors associated with 28-day mortality (Table S3). We then assessed temperature rhythm parameters and 28-day mortality (Table 3).

Due to strong collinearities between the sepsis severity (SOFA score) and therapeutics (dialysis, sedation, vasopressors, and hydrocortisone), we built different models of multivariate analysis. Moreover, dialysis (which uses an extracorporeal circuit) was strongly associated with a low amplitude of temperature (Table 2), but it was also associated with mortality, and it mitigated the association between amplitude and mortality. Our final model (model 4) thus excluded dialysis in the adjusted analysis. Multivariate Cox regression analysis showed that lower mesor (adjusted hazard ratio 0.50, 95% CI 0.28 to 0.90; *p* = 0.02) and higher amplitude (adjusted hazard ratio 5.48, 95% CI 1.66 to 18.12; *p* = 0.005) of temperature were associated with 28-day mortality (Table 3).

**Figure 3:**
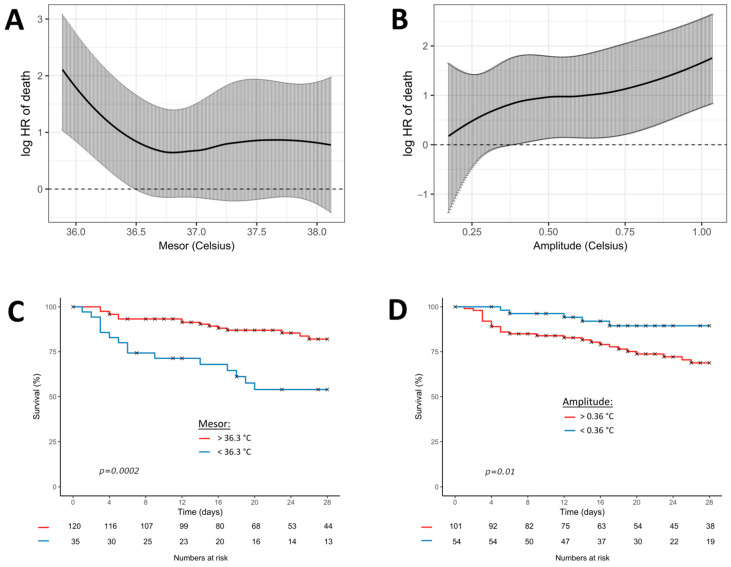
Cox regression model fitting the association between temperature (mesor and amplitude) and 28-day mortality. Black curves represent the estimates of 28-day mortality (multivariate model 4) according to the continuous values of mesor (**A**) and amplitude (**B**). Grey bars represent the 95% confidence intervals. The graphs below represent the Kaplan-Meier curves of the 28-day mortality according to the most significant threshold of mesor (**C**) and amplitude (**D**). *p*-values result from the log-rank analysis between groups. Red curves represent the patients above the most significant threshold and the blue curves below the most significant threshold.

Figure 3 shows the Cox regression model, fitting the continuous association between mesor or amplitude and the log relative hazard of 28-day mortality. The best significant thresholds of the circadian parameters for predicting 28-day mortality were 36.3 °C for the mesor (*p* < 0.001) and 0.36 °C for the amplitude (*p* < 0.001).

## 4. Discussion

In this description of the rhythm of the body temperature in septic shock patients, we found that lower level of temperature and higher amplitude of variation appear to be associated with 28-day mortality.

These results confirm previous studies, which show that, between 10% and 20% of patients with sepsis present, a “hypothermia” (variably defined as ≤35.5–36.5 °C) leads to a mortality rate almost twice that of pyretic patients [27]. Another observational study by Drewry et al. described an independent association between early hypothermia and persistent lymphopenia in patients with sepsis. In this retrospective study, the hypothermic cohort was characterized by significant excess mortality compared with non-hypothermic controls (50% vs. 25% at day 28) [19]. In mammals, elevated body temperatures generally promote the activation, function, and delivery of immune cells. Conversely, hypothermia reduces these processes and thus participates in immuno-paralysis of the host response [28].

We demonstrated an association between high amplitude and mortality. One of the first ICU studies to focus on circadian rhythm, via the evaluation of the CBT, reported that 80% of the patients had significant circadian rhythms with erratic acrophases (peak time), but they had normal amplitudes; nevertheless, there was a tendency for the amplitude of the temperature rhythm to be greater in non-survivors than in survivors [9]. Amplitude changes might result from a clock activation in this specific context of infection, which may involve host response and circadian clocks. Thus, high amplitudes might be the marker of initial sepsis severity and are associated with a profound initial hyper-inflammatory response and a disruption of the immunity homeostasis, as described in sepsis [29].

Besides, many factors, such as gender or therapeutics, were, in our study, associated with rhythm patterns of CBT. As expected, acetaminophen and dialysis were associated with different temperature changes. Acetaminophen is a well-known central antipyretic, but its mechanisms remain incompletely understood, and many other effects were described, including hemodynamic changes in ICU septic patients [30]. In our study, acetaminophen was associated with a lower period that may be explained by a discontinuous administration (every 6 h in our protocol). However, effects may be bidirectional, and the direct central effect of acetaminophen on the temperature may not be excluded. Dialysis (which uses an extracorporeal system) may cool the blood and favors hypothermia. However, we demonstrated an association with lower amplitude. The external heaters of the dialysis circuit target a constant temperature and thus may explain these results. Moreover, dialysis was, in our study, logically associated with 28-day mortality, but it also had strong collinearity with sepsis severity (including SOFA or sedation, as well as vasoactive drugs). Dialysis tends to lower the effect of temperature (especially the amplitude) on 28-day mortality, which is why we built a model (model 4) without this variable in our adjusted analysis. The association of the amplitude and 28-day mortality may not be related to the dialysis because they had inverse effects (high amplitude was associated with mortality; dialysis was associated with mortality and lower amplitude).

Interestingly, we found an association between gender and the period of temperature. The influence of gender on immunity and response to infection is now well described [31], and we have also previously shown the correlation between gender, infection, and circadian rhythm in a murine model of bacterial infection [32].

We performed mathematical modeling (Fourier transform) to approach the most precise actual period of each patient. Interestingly, we found that, whereas some patients had a period around 24 h, more than half exhibited a faster rhythm with a period that could drop down to 4 h to a complete cycle of temperature. However, we did not demonstrate any association between period and mortality. This might suggest that rhythm acceleration in the case of sepsis could be a marker of good rhythmic adaptation when necessary. Indeed, a different rhythm from 24 h, usually interpreted as abnormal, could result from a physiological adaptation during the ICU stay. It may be interesting to assess, in future prospective studies, the evolution of the period as a prognostic tool in sepsis. In most of the studies on CBT, the authors were interested mainly in the presence or absence of a circadian rhythm (i.e., a rhythm with a period of 24 h) and used methods much more basic, relying for example on the hour of the maximum peak of CBT by comparing it to the reference values. The low point of CBT can be used to identify the end of the circadian night, and, in ICU patients, the degree of CBT displacement from its customary early morning locus correlates with illness severity [9,10,33].

Evidence suggests that body temperature pattern analysis might lead to meaningful clinical information, which is also described in terms of the diagnosis of sepsis. Papaioannou et al. studied the temperature patterns using linear discriminant analysis and cluster analysis by extracting wavelet features in critically ill patients with suspected ICU-acquired infections [34]. They extracted different wavelet features from the temperature pattern among the three groups (systemic inflammatory response syndrome (SIRS), sepsis, and septic shock) and found statistically significant outcomes, with decreased variability in the more severe groups. Even if the temperature pattern seems to be more useful than a single value at admission, these variables are more complex to compute than the period, amplitude, and mesor of the temperature signal that we used in the present study.

The causality between circadian disruption and the immune response is unclear. In ICU, sepsis and its severity are both associated with circadian disruption [6]. We also demonstrated an association between circadian disruption and ICU-acquired infection in trauma patients [35]. However, in vitro and animal data evidenced the negative effect of inflammation on the molecular clock [5,36]. Conversely, circadian disruption and clock gene inhibition dramatically affect immune functions and response to infection [5,37]. The molecular clock contributes to immune homeostasis; the deregulation of this clock could thus break this homeostasis and participate in an unadapted inflammatory balance, affecting immune functions. Indeed, many animals’ experimental studies have found that the lethality induced by lipopolysaccharide (LPS), or recombinant human tumor necrosis factor (TNF-alpha), varied significantly throughout the day, depending on the time of administration, emphasizing an important link with the circadian rhythm [38].

It is importance to note that altered phase positions in ICU may result from abnormal temporal cues in its environment, which can cause the desynchronization of the circadian pacemaker. Indeed, studies have suggested that a lot of zeitgebers (external or environmental cues that entrain the clock) are abnormal in the ICU [8,39]. Light patterns appear to be different for patients in the ICU compared with normal control subjects [40]. Besides, sleep and wakefulness are highly fragmented and generally evenly distributed over the 24 h of the day [41]. Sleep deprivation has been demonstrated to be associated with lower temperature levels and may be involved in the circadian disruption of CBT [42]. Food intake was also found to affect CBT [43]. Our patients were fed continuously by enteral tube, 24 h a day, which is not physiological and may participate in circadian disruption. Lastly, ICUs represent a noisy environment and could be involved in the disruption of the circadian rhythm via sleep abnormalities [21].

This study has several limitations. This is a monocenter study, and patients may have been exposed to standardized protocols specific to our center that affect temperature rhythm and that have not been identified by the study. The measurement of the temperature was either central or by tympanic devices, which may lead to differences in temperature reading. However, the circadian parameters (Table S2) were not associated with the technique of measurement. The measurements were taken every 2 or 4 h, not allowing a very fine fitting of the period, as recordings could accomplish every minute. The mathematical model tries to fit the best period using sinusoid laws. It is, thus, difficult to know if the CBT follows a rhythmic pattern or if it is random variations. Any interventions that may affect CBT similar to a single shot of acetaminophen may simulate a rhythm not generated by the internal clock. Besides, not all factors that may influence CBT have been considered, such as blankets, clothes, or muscular activity. Additionally, it would be interesting to compare CBT with non-septic ICU patients.

## 5. Conclusions

Our study showed that early temperature pattern disruptions, lower mesor, and higher amplitude were associated with mortality in this cohort of septic shock. In the age of artificial intelligence, integrating such data in an automated scoring alert could help to identify high-risk patients during septic shock. These results need to be confirmed, and it would be interesting to assess by which mechanism this adaptation occurs—genomic, transcriptomic, or metabolic adaptation.

## Figures and Tables

**Table 1:** Patient characteristics of the cohort. Qualitative data are expressed as number and percentage, *n* (%). Quantitative data are expressed in median and interquartile, median [IQR]. BMI: body mass index; ICU: intensive care unit.

Characteristics	Cohort (*n* = 162)
Age (years)	65 [56–74]
Gender (Women)	93 (57.4)
Weight (kg)	75 [66–86]
BMI (kg/m^2^)	27 [23–30]
Smoke history, *n*(%)	65 (40.1)
Betablocker use, *n*(%)	56 (35.4)
Comorbidities, *n* (%)	
*Diabetes*	51 (31.5)
*Chronic pulmonary disease*	36 (22.2)
*Addiction*	24 (14.8)
*Renal failure*	23 (14.2)
*Non-resolutive cancer*	23 (14.2)
*Ischemic heart disease*	17 (10.5)
*Immunosuppressed*	17 (10.5)
*Severe neurological disorder*	5 (3.1)
*Cirrhosis*	4 (2.5)
*Congestive heart failure*	2 (1.2)
SOFA at 24 h (score)	11 [9–13]
IGS II at admission (score)	67 [48–83]
Procalcitonine at admission (ng/mL)	19 [3–45]
Tympanic measure, *n* (%)	69 (42.6)
Fever at day 2, *n* (%)	56 (34.6)
Therapeutics at day 2, *n* (%)	
*Extra-renal remplacement*	37 (22.8)
*Sedation*	110 (67.9)
*Vasopressor support*	125 (77.2)
*Curare*	17 (10.5)
*HSHC*	92 (56.8)
*Steroide*	12 (7.4)
*Acetaminophen*	27 (16.7)
Mechanical ventilation, *n* (%)	141 (87.0)
Duration of mechanical ventilation (days)	6 [3–13]
ICU stay length (days)	9 [5–18]
ICU death, *n* (%)	38 (23.5)
Hospital death, *n* (%)	49 (30.2)
Site of infection, *n* (%)	
*Lung*	75 (46.3)
*Urinary tract*	29 (17.9)
*Intra-abdominal*	24 (14.8)
*Skin*	14 (8.6)
*Bone/Joint*	4 (2.5)
*Blood*	3 (1.9)
*Teeth*	2 (1.2)
*Systemic infection (malaria)*	1 (0.6)
*Meningitidis*	1 (0.6)
*Unknown*	9 (5.6)
Bacteremia, *n* (%)	72 (44.4)

**Table 2:** Multivariate analysis assessing the associations between patient characteristics and the three parameters of the temperature (Period, Mesor, and Amplitude). The analyses were performed using the general linear model. Results expressed the beta estimates (Coefficient) with their standard errors (SE) and the odds ratios (OR) with their 95% confidence intervals (95% CIs).

Multivariate	Coefficient	SE	OR [95% CI]	*p*-Value
Period				
Gender (Women)	−2.164	0.995	0.11 [0.02–0.81]	0.03
Acetaminophen	−4.334	1.354	0.013 [0.001–0.19]	0.002
Mesor				
Smoke history	−0.194	0.143	0.82 [0.62–1.09]	0.18
SOFA at 24 h	−0.051	0.025	0.95 [0.91–0.99]	0.046
Procalcitonin (ng/mL)	0.004	0.001	1.004 [1.001–1.006]	0.005
Intra-abdominal	−0.345	0.229	0.71 [0.45–1.11]	0.13
Dialysis	0.010	0.186	1.01 [0.70–1.45]	0.96
Hydrocortisone	−0.500	0.160	0.61 [0.44–0.83]	0.002
Amplitude				
Dialysis	−0.495	0.156	0.61 [0.45–0.83]	0.002

**Table 3:** Survival analysis assessing mortality on day 28 and circadian rhythm parameters of the temperature (Period, Mesor, and Amplitude). The analyses were performed using the Cox regression model to estimate hazard ratios (HR) and their 95% confidence intervals (95%CIs). Analysis was performed with the period as a categorial variable (based on two groups divided on the median of 20.8 h, which corresponds to a standard circadian rhythm for patients with period above the median) or as a continuous variable. Model 1 is an adjusted analysis on the SOFA score. Model 2: Model 1 + dialysis, sedation, vaso-active drugs use, and hydrocortisone use. Model 3: Model 2 + the three circadian rhythm parameters together (period, mesor, and amplitude). Model 4: Model 3 without dialysis.

	Low Period (Categorial)		Period (Continuous)		Mesor (Continuous)		Amplitude (Continuous)	
	HR [95% CI]	*p*	HR [95% CI]	*p*	HR [95% CI]	*p*	HR [95% CI]	*p*
Univariate	1.04 [0.52–2.08]	0.91	1.00 [0.94–1.06]	0.99	0.49 [0.29–0.80]	0.004	4.00 [1.43–11.18]	0.008
Model 1	1.16 [0.58–2.34]	0.67	0.99 [0.93–1.05]	0.64	0.54 [0.32–0.92]	0.02	4.83 [1.66–14.01]	0.004
Model 2	0.95 [0.47–1.92]	0.91	0.98 [0.92–1.05]	0.58	0.55 [0.30–1.00]	0.05	2.51 [0.84–7.53]	0.10
Model 3	0.30 [0.06–1.51]	0.14	0.95 [0.89–1.02]	0.17	0.51 [0.28–0.93]	0.03	4.03 [1.17–13.93]	0.03
Model 4	0.34 [0.07–1.65]	0.18	0.95 [0.88–1.02]	0.13	0.50 [0.28–0.90]	0.02	5.48 [1.66–18.12]	0.005

## Data Availability

Not applicable.

## Supplementary Materials

The following supporting information can be downloaded at: https://www.mdpi.com/article/10.3390/biology12050638/s1, Table S1: Description of the causal infectious agents documented during the ICU stay; Table S2: Univariate analysis assessing the associations between patient characteristics and the three parameters of the temperature (Period, Mesor, and Amplitude); Table S3: Univariate survival analysis assessing mortality at day 28, patient characteristics, infection characteristics, and therapeutics.
