# Supplementary material for: Changes in Body Temperature Patterns Are Predictive of Mortality in Septic Shock: An Observational Study
Source: Biology (Basel). 2023 Apr 22;12(5):638. doi: 10.3390/biology12050638 (PMC10215947; doi:10.3390/biology12050638)
Supplement: Supplementary file 1 [file biology-12-00638-s001.zip › biology-2327630-supplementary.pdf]

Table S1: Description of the causal infectious agents documented during the ICU stay. Qualitative data are expressed as number and percentage, n (%).

**Main causal agent**		
	Enterobacteria, group I	28 (21.9)
	Streptococcus group	25 (19.5)
	Staphylococcus group	20 (15.6)
	Enterobacteria, group II	12 (9.4)
	Enterobacteria, group III	8 (6.2)
	Pseudomonas aeruginosa	8 (6.2)
	Enterococcus group	6 (4.7)
	Haemophilus	4 (3.1)
	Clostridium	4 (3.1)
	Stenotrophomonas	3 (2.3)
	Bacteroides	3 (2.3)
	Moraxella	2 (1.6)
	Legionella	2 (1.6)
	Pneumocystis	1 (0.8)
	Neisseria meningitidis	1 (0.8)
	Achromobacter	1 (0.8)
	Not found	34 (21.0)
Multiple agents		54 (33.3)
**Second agent**		
	Streptococcus group	13 (24.1)
	Enterobacteria, group I	9 (16.7)
	Enterobacteria, group II	8 (14.8)
	Staphylococcus group	5 (9.3)
	Enterococcus group	4 (7.4)
	Pseudomonas aeruginosa	4 (7.4)
	Enterobacteria, group III	3 (5.6)
	Pneumocystis	2 (3.7)
	Stenotrophomonas	2 (3.7)
	Achromobacter	1 (1.8)
	Clostridium	1 (1.8)
	Fusobacterium	1 (1.8)
	Haemophilus	1 (1.8)

Table S2: Univariate analysis assessing the associations between patient characteristics and the three parameters of the temperature (Period, Mesor, and Amplitude). The analyses were performed using the general linear model. Results expressed the coefficient estimates (beta) with the standard error (SE). BMI: body mass index.

	**Period at day 2**			**Mesor at day 2**			**Amplitude at day 2**		
	Coefficient	SE	p	Coefficient	SE	p	Coefficient	SE	p
Age (years)	0.039	0.036	0.28	-0.006	0.004	0.16	-0.001	0.002	0.53
Sex (Women)	-1.932	0.968	0.048	0.126	0.122	0.30	-0.062	0.047	0.19
BMI (kg/m2)	0.098	0.073	0.18	0.006	0.009	0.49	0.003	0.004	0.46
Smoke history	-1.037	0.988	0.30	-0.340	0.121	0.005	0.013	0.048	0.79
SOFA at 24 hours	0.266	0.155	0.09	-0.053	0.019	0.006	0.004	0.008	0.56
Procalcitonine (ng/mL)	0.006	0.012	0.64	0.003	0.001	0.047	-0.001	0.001	0.97
Betablocker use	-0.310	1.029	0.76	-0.026	0.129	0.84	0.008	0.049	0.86
Comorbidities									
Diabetes	1.001	1.045	0.34	0.045	0.131	0.73	-0.061	0.051	0.23
Chronic pulmonary disease	-0.440	1.174	0.71	0.157	0.146	0.28	-0.023	0.057	0.68
Renal failure	0.995	1.389	0.47	-0.204	0.173	0.24	0.019	0.068	0.78
Non-resolutive cancer	-0.383	1.392	0.78	-0.224	0.173	0.20	-0.023	0.068	0.73
Ischemic heart disease	-1.461	1.550	0.34	-0.165	0.194	0.39	-0.112	0.075	0.14
Immunosuppressed	-2.054	1.635	0.21	0.042	0.205	0.84	-0.034	0.080	0.67
Severe neurological disorder	4.107	2.723	0.13	-0.322	0.342	0.35	0.006	0.134	0.96
Cirrhosis	1.980	3.060	0.52	-0.461	0.381	0.23	-0.143	0.149	0.34
Congestive heart failure	5.513	4.282	0.20	-0.673	0.535	0.21	-0.124	0.209	0.55
Site of infection									
Lung	reference			reference			reference		
Urinary tract	1.135	1.342	0.40	-0.019	0.168	0.91	0.028	0.066	0.67
Intra-abdominal	1.315	1.443	0.36	-0.458	0.180	0.01	-0.019	0.071	0.79
Skin	-0.477	1.757	0.79	-0.079	0.220	0.72	0.108	0.087	0.21
Bone/Joint	-3.583	3.086	0.25	0.304	0.386	0.43	0.203	0.152	0.18
Blood	-7.624	3.539	0.033	-0.152	0.443	0.73	-0.164	0.174	0.35
Teeth	3.661	4.305	0.40	0.647	0.538	0.23	0.223	0.212	0.29
Systemic infection (malaria)	5.382	6.045	0.37	-0.248	0.756	0.74	-0.143	0.298	0.63
Meningitidis	4.311	6.045	0.48	0.012	0.756	0.99	-0.155	0.298	0.60
Unknown	-0.387	2.126	0.86	-0.131	0.266	0.62	0.046	0.105	0.66
Bacteriemia	0.519	0.975	0.60	-0.224	0.121	0.06	0.057	0.047	0.23
Tympanic measure	-0.613	0.990	0.54	0.123	0.123	0.32	-0.080	0.048	0.10

Table S3: Univariate survival analysis assessing mortality at day 28, patient characteristics, infection characteristics, and therapeutics. The analyses were performed using the Cox regression model to estimate hazard ratios (HR) and their 95% confidence intervals (95%CIs). BMI: body mass index; NMBA: neuromuscular blocker agent.

	**HR [95% CI]**	**p**
Age (years)	1.02 [0.99-1.05]	0.18
Sex (Women)	1.07 [0.53-2.17]	0.85
BMI (kg/m2)	0.99 [0.94-1.05]	0.89
Smoke history	1.13 [0.56-2.28]	0.73
SOFA at 24 hours	1.17 [1.06-1.29]	0.002
Procalcitonin (ng/mL)	0.99 [0.97-1.01]	0.20
Betablocker use	1.13 [0.54-2.38]	0.73
Comorbidities		
Diabetes	0.58 [0.25-1.35]	0.21
Chronic pulmonary disease	1.03 [0.45-2.38]	0.94
Renal failure	0.17 [0.02-1.27]	0.09
Non-resolutive cancer	1.76 [0.76-4.06]	0.19
Ischemic heart disease	1.31 [0.46-3.73]	0.62
Immunosuppressed	0.59 [0.14-2.47]	0.47
Severe neurological disorder	0.95 [0.13-6.96]	0.96
Cirrhosis	0.00 [0.00-Inf.]	0.99
Congestive heart failure	3.27 [0.44-24.1]	0.24
Site of infection		
Lung	reference	
Urinary tract	0.62 [0.21-1.85]	0.39
Intra-abdominal	0.97 [0.36-2.62]	0.94
Skin	0.88 [0.26-3.02]	0.84
Bone/Joint	0.00 [0.00-Inf.]	0.99
Blood	0.00 [0.00-Inf.]	0.99
Teeth	2.15 [0.29-16.19]	0.46
Malaria	3.20 [0.43-24.16]	0.26
Meningitidis	0.00 [0.00-Inf.]	0.99
Unknown	0.45 [0.21-1.85]	0.44
Bacteriemia	0.79 [0.39-1.60]	0.51
Treatment		
Acetaminophen	1.22 [0.50-2.97]	0.66
Dialysis	5.56 [2.74-11.27]	<0.001
Sedation	7.27 [1.74-30.44]	0.007
NMBA	1.97 [0.81-4.78]	0.13
Vaso-active drugs	9.78 [1.33-71.68]	0.02
Hydrocortisone	3.45 [1.43-8.4]	0.006
Steroids	0.94 [0.23-3.95]	0.94
